# Intermediate-term outcomes of laparoscopic pectopexy and vaginal sacrospinous fixation: a comparative study

**DOI:** 10.1590/S1677-5538.IBJU.2019.0103

**Published:** 2019-01-29

**Authors:** Bahar Sariibrahim Astepe, Aybike Karsli, Işil Köleli, Orhan Seyfi Aksakal, Hasan Terzi, Ahmet Kale

**Affiliations:** 1 Department of Obstetrics and Gynecology Kocaeli Derince Training and Research Hospital Kocaeli Turkey Department of Obstetrics and Gynecology, Kocaeli Derince Training and Research Hospital, Kocaeli, Turkey;; 2 Department of Obstetrics and Gynecology Inönü University Medicine Faculty Malatya Turkey Department of Obstetrics and Gynecology, Inönü University Medicine Faculty, Malatya, Turkey;; 3 Urogynecology Clinics Zekai Tahir Burak Training and Research Hospital Ankara Turkey Urogynecology Clinics, Zekai Tahir Burak Training and Research Hospital, Ankara, Turkey

**Keywords:** Pelvic Organ Prolapse, Hand-Assisted Laparoscopy, Treatment Outcome

## Abstract

**Objective:**

To compare the intermediate-term follow-up results of laparoscopic pectopexy and vaginal sacrospinous fixation procedures.

**Materials and Methods:**

Forty-three women who had vaginal sacrospinous fixations(SSF) using Dr. Aksakal’s Desta suture carrier and 36 women who had laparoscopic pectopexies were re-examined 7 to 43 months after surgery. The PISQ-12 and P-QOL questionnaires were answered by all of the women.

**Results:**

The apical descensus relapse rates did not differ between the groups (14% in the SSF vs. 11.1% in the pectopexy group). The de novo cystocele rates were higher in the SSF group (25.6% in the SSF vs. 8.3% in the pectopexy group). There were no significant differences in the de novo rectocele numbers between the groups. The treatment satisfaction rates were high in both groups (93% in the SSF vs. 91.7% in the pectopexy group), which was not statistically significant. Moreover, the postoperative de novo urge and stress urinary incontinence rates did not differ; however, the postoperative sexual function scores (PISQ-12) (36.86±3.15 in the SSF group vs. 38.21±5.69 in the pectopexy group) were better in the pectopexy group. The general P-QOL scores were not significantly different between the surgery groups.

**Conclusion:**

The vaginal sacrospinous fixation maintains its value in prolapse surgery with the increasing importance of native tissue repair. The new laparoscopic pectopexy technique has comparable positive follow-up results with the conventional sacrospinous fixation procedure.

## INTRODUCTION

A pelvic organ prolapse (POP) is defined as a herniation of the pelvic organs to or beyond the vaginal walls, and can significantly affect a woman’s daily activities and sexuality. The surgical approaches to the POP treatment vary, including vaginal, open abdominal, laparoscopic, and robotic methods. The decision regarding the surgical route depends on the surgeon’s experience, the need for repairing other pelvic organ defects, and the coexistence of urinary incontinence.

In 2016, the US Food and Drug Administration (FDA) reclassified the use of surgical mesh for transvaginal POP surgery as a class 3 procedure (i.e., high risk) (1). Since this FDA reclassification, native tissue repair in vaginal surgery and laparoscopic procedures has been gaining more importance. In vaginal surgery, native tissue repair eliminates the mesh-related complications, such as mesh erosion and infection. This is particularly important for women at a high risk of mesh erosion (e.g., women who smoke, are immunosuppressed, or have uncontrolled diabetes mellitus).

With the promising advantages of laparoscopic surgery, such as shorter hospitalization, shorter recovery time, less postoperative pain, early mobilization, and fewer scars (2), minimal invasive surgery for POP treatment has become more significant. In 1993, Joshi VM. described a new technique for uterine suspension to pectineal ligament bilaterally with mersile tape through a Cherney incision (3). In 2010, Banerjee and Noe described a laparoscopic pectopexy operation for obese patients using the iliopectineal ligament for the vaginal vault or cervical stump suspension (4). To our best knowledge, we have yet to find literature in the area of postoperative results comparing laparoscopic pectopexy (LPP) and vaginal sacrospinous fixation procedures (VSSF). In the present study, we aimed to compare the intermediate-term outcomes of the pectopexy technique and conventional sacrospinous fixation in our clinic.

## MATERIALS AND METHODS

This retrospective observational study was based on the postoperative results of women who had undergone uterovaginal or vaginal vault prolapse surgery. Forty-three women who had VSSF and 36 women who had LPP between January 2014 and June 2018 at the S.B.U Kocaeli Derince Education and Research Hospital Obstetrics and Gynecology Clinics were gynecologically re-evaluated between 15 June and 30 December 2018.

Preoperatively, all of the patients provided urogynecological histories and underwent routine physical examinations, cough stress tests, perineal ultrasonography, and Bonney tests. The POP (apical, anterior and posterior wall prolapsus) was staged according to the Pelvic Organ Prolapse Quantification (POP-Q) system. Those patients with stage 2 or greater uterovaginal/vaginal cuff prolapses according to the POP-Q system underwent surgery (vaginally or laparoscopically) were included in the study. Patients with stage 2 uterine / vaginal cuff prolapsus were recommended to have surgery if they had complaints of bulging symptoms. Young women with fertility desire and who had had hysteropexy procedures were not included in the study. Women who had had surgery for malignancy suspicion or pelvic inflammatory disease were not included in the study. Those patients with stress urinary incontinence and positive cough stress tests underwent anti-incontinence surgery (transobturator tape or tension free vaginal tape) along with the POP surgery. The hospital database was evaluated for any perioperative and postoperative complication records. All of the methods and definitions used in the study conformed with the standards recommended by the International Urogynecological Association and the International Continence Society (5). Ethical approval for this study was obtained from the S.B.U Kocaeli Derince Training and Research Hospital Management and Training Commission. The clinical trials registration ID is NCT03663959.

All of the patients received telephone calls and those patients that could be reached at phone were invited for a gynecological re-examination. All but one patient in the vaginal surgery group came in for a gynecological check. All patients gave written informed consent for the scientific use of their evaluation results, operation videos and images in this study. We conducted a telephone interview with the one patient not agreeing to come for the check, and we learned that she had had a relapse 6 months after the operation and she was unsatisfied with the surgery. In the postoperative re-evaluation between 15 June and 30 December 2018, all of the women were examined in the lithotomy position for apical, anterior, and posterior compartment descensus. Stage 2 or greater apical descensus or a cystocele or rectocele according to the POP-Q system were accepted as postoperative relapses. All of the patients answered the Pelvic Organ Prolapse / Urinary Incontinence Sexual Questionnaire (PISQ-12) and the Prolapse Quality of Life (P-QOL) questionnaire. The Turkish validations of the PISQ-12 and P-QOL were conducted by Cam et al. (6, 7). The PISQ-12 is a self-administered questionnaire that evaluates the sexual function of women with pelvic organ prolapse or urinary incontinence. Higher scores show good sexual functioning of women. The P-QOL evaluates the impact of the urogenital prolapsus on the quality of life in women. A high total score indicates a worsening of quality of life of women with pelvic organ prolapsus.

All of the women were asked about de novo urge urinary incontinence and de novo stress urinary incontinence. In addition, each patient’s satisfaction with the surgery was asked and recorded.

**Figure 1:**
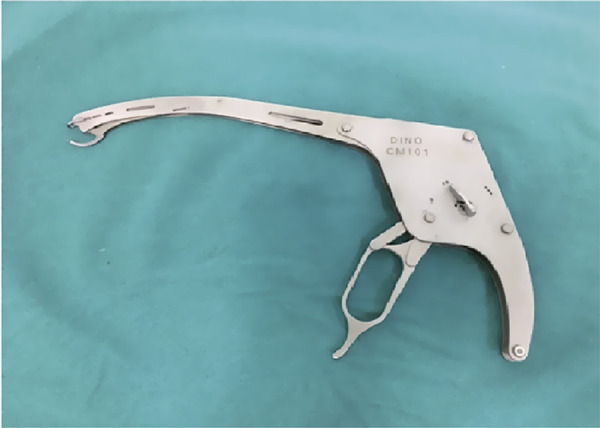
Dr. Aksakal's Desta suture carrier.

The vaginal surgery group underwent a right sacrospinous fixation using Dr. Aksakal’s Desta suture carrier (Figure-1) with two permanent sutures, which combined the sacrospinous ligament and vaginal cuff fascia. The Desta suture carrier was developed for deep pelvic surgery, and the suture depth can be easily adjusted. After the vaginal hysterectomy, under the vaginal cuff mucosa, we created a tunnel through the spinous process with a straight tool. After passing the rectovaginal pillars, the perirectal space was entered, and the ischial spine was palpated. With the help of an index finger placed on the spinous process, we placed two permanent sutures 1.5-2 cm medial onto the spinous process on the sacrospinous ligament and iliococcygeus muscle complex using the Desta suture carrier. Next, the permanent sutures were combined with the pubocervicovaginal and rectovaginal fascia under the vaginal cuff mucosa. Those patients with stress urinary incontinence underwent transobturator tape or tension free vaginal tape procedures. Those patients with lateral defect cystoceles underwent paravaginal repairs that were performed using late absorbable sutures that combined the arcus tendineus fasciae pelvis and pubocervi covaginal fascia.

In vaginal surgery, in stage 2 and over apical prolapsus there is a great endopelvic fascia defect that cause prolapsus not only in apical compartment but also in the anterior and posterior compartments. If there is Stage 2 and over cystocele or rectocele according to the POP-Q we add anterior or posterior colporraphies to the operation.

**Figure 2:**
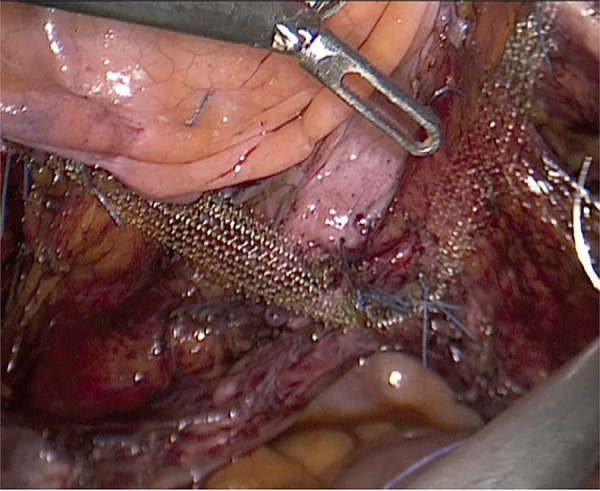
The mesh is fixated to the bilateral ileopectineal ligaments and anterior part of the vaginal cuff.

The laparoscopy group underwent pectocolpopexy procedure. After entering into the abdominal cavity with 10mm (EndoEthicon) and 5 mm trocars, sufficient intraabdominal pressure (14 mm Hg) was achieved. First, the peritoneal layer above and lateral to the bladder was opened parallel to the round ligament toward the pelvic side wall on the right side. Then, with the guidance of the obliterated umbilical artery, lateral to the obliterated umbilical artery and medial to the external iliac vein, the iliopectineal ligament was located. At this point, a segment of approximately 3-4 cm2 was formed, exposing the iliopectineal (Cooper’s) ligament. In this area, behind the obliterated umbilical artery, the obturator nerve could be seen. The same area on the left side was prepared using the same steps. Then, the anterior part of the vaginal cuff was prepared for the mesh fixation. Bilaterally, the ends of a polypropylene monofilament mesh (1.5 x 15 cm) were fixed to the iliopectineal ligament with nonabsorbable polypropylene or polyester sutures (Prolene & Ethibond Excel; Ethicon, Somerville, NJ, USA) (Figure-2). The vaginal cuff was elevated to the POP-Q level 0-1 with the help of a vaginal sponge, and the mesh was fixed anteriorly to the vaginal cuff with nonabsorbable polypropylene sutures. Finally, the mesh was embedded under the peritoneum with a continuous monofilament absorbable suture (Vicryl; Ethicon, Johnson & Johnson, Bridgewater, NJ, USA).

In L / S pectopexy surgery, after apical suspension we evaluate anterior and posterior vaginal walls. If there is Stage 2 and over cystocele or rectocele according to the POP-Q, we add anterior or posterior colporraphies to the operation.

### Statistics

The sample size calculation was done with the G Power 3.1 software. For the comparison of the categorical variables between the groups, with the significance level of α:0.05, the effect size 0.5 and the statistical power of 0.80, the required total sample size was 52.

The IBM SPSS Statistics for Windows version 21.0. (IBM Corp., Armonk, NY, USA) was used for the statistical analysis. The continuous variables were expressed as the mean±standard deviation, and the categorical variables were expressed as the number and percentage. The Mann-Whitney U test and independent sample t-tests were used to compare the quantitative variables, while the chi-squared test and Fischer’s exact test were used to compare the categorical variables. The statistical significance was defined as p <0.05.

## RESULTS

Forty-three patients underwent VSSFs, and 36 patients underwent LPPs. Forty patients in the vaginal surgery group and 18 patients in the laparoscopic surgery group underwent hysterectomies (vaginal hysterectomy or total laparoscopic hysterectomy). Of the 79 patients, 21 patients had vaginal vault prolapses (three in the vaginal surgery group and eighteen in the laparoscopy group) (Table-1). Thirteen patients in the vaginal surgery group and three patients in the laparoscopy group underwent concomitant anti-incontinence surgery.

**Table 1:** Surgical procedures performed in the vaginal and laparoscopic surgery groups.

Procedure	Vaginal Sacrospinous Fixation group (n: 43)	Laparoscopic Pectopexy Group (n: 36)
Vaginal Hysterectomy	40 (93%)	-
Sacrospinous fixation	43 (100%)	-
Anterior colporrhaphy	28 (65.1%)	2 (5.6%)
Posterior colporrhaphy	23 (53.5%)	2 (5.6%)
Salphenjectomy	8 (18.6%)	-
Perineoplasty	17 (39.5%)	2 (5.6%)
Transobturator tape	11 (25.6%)	3 (8.3%)
Tension free vaginal tape	2 (4.7%)	-
Paravaginal repair	10 (23.3)	5 (13.9%)
Laparoscopic hysterectomy	-	18 (50%)
Laparoscopic pectocolpopexy	-	36 (100%)
Bilateral salphingoopherectomy	-	21 (58.3%)

The mean ages of the patients were 58.95±8.33 and 60.03±9.05 in the vaginal surgery and laparoscopy group respectively, which was not statistically significant. The number of births of the patients in the surgery groups was not different. There were no significant differences in the duration of the follow-up period between the surgery groups. The vaginal surgery patients were examined 7 to 43 (min-max) months after the surgery with a mean of 17.09±10.29 months, and the laparoscopic surgery patients were examined 7 to 30 (min-max) months after surgery with a mean of 13.06±6.35. Three patients in the laparoscopy group had repeat surgery because of a recurrent prolapse; two of them had SCP and 1 had previously had VSSF (Table-2).

**Table 2:** Patient characteristics and previous pelvic surgery histories in the vaginal and laparoscopic surgery groups.

Parameter	Vaginal Sacrospinous fixation group (n: 43)	Laparoscopic Pectopexy group (n: 36)	p
Age (years)	58.95±8.33	60.03±9.05	0.585a
Mean body mass index (kg / m^2^)	28.98±3.48	29.51±3.29	0.492a
Parity	4.02±2.01	3.72±1.75	0.613b
Follow-up (month)	17.09±10.29	13.06±6.35	0.165b
History of pelvic surgery, n (%)	5 (11.6%)	21 (58.3%)	0.000c
Abdominal hysterectomy + bilateral salpingoophorectomy	2	11	
Abdominal hysterectomy	1	1	
Vaginal Hysterectomy	-	6	
Tubal ligation	1	1	
Tubal ligation + unilateral salpingoophorectomy	1	-	
Cesarean section	-	2	
Sacrocolpopexy	-	2	
Sacrospinous fixation	-	1	

^a^ Independent samples t-test; ^b^ Mann-Whitney U test; ^c^ Pearson Chi-squared test

The patient’s hospital records were evaluated for perioperative and postoperative complications. One patient had ureteral kinking in the vaginal surgery group. She underwent ureteral catheterizations four times postoperatively, and she is now in urology follow-up. One patient in the laparoscopy group had a bladder injury. Another patient in the laparoscopy group had mesh erosion at the vaginal apex, which was surgically extracted. The woman with mesh erosion was 50 years old, she had had a vaginal hysterectomy two years earlier and a laparoscopic sacrocolpopexy one year earlier (Table-3).

**Table 3:** Complications related to surgery groups.

	Vaginal sacrospinous fixation group (n: 43)	Laparoscopic pectopexy group (n: 36)
Ureteral kinging (n)	1	
Bladder injury (n)		1
Mesh erosion (n)		1

There were six apical descensus relapse cases in the vaginal surgery group and four cases in the laparoscopy group, although these numbers were of no statistical significance. The number of women with de novo central or lateral defect cystoceles in the vaginal surgery group was significantly higher than the number of women in the laparoscopy group (Table-4). Those women who underwent VSSFs had more de novo cystoceles than the women who underwent the LPPs. Moreover, there was no significant difference in the de novo rectocele numbers between the vaginal and laparoscopic surgery groups. The treatment satisfaction rates were high in both groups (93% in the vaginal surgery and 91.7% in the laparoscopy groups), but this was not statistically significant.

**Table 4:** Follow-up results.

	Vaginal sacrospinous fixation group	Laparoscopic pectopexy group	p
Number of patients	43	36	
Apical descensus relapse (number of patients / all patients)	6 (14%)	4 (11.1%)	0.748^d^
De novo central or lateral defect cystocele (number of patients / all patients)	11 (25.6%)	3 (8.3%)	0.046^c^
De novo rectocele (number of patients / all patients)	2 (4.7%)	2 (5.6%)	1.000^d^
De novo stress urinary incontinence	1 (2.3%)	2 (5.6%)	0.589^d^
De novo urge incontinence	3 (7%)	0 (0%)	0.246^d^
Satisfied with surgery (number of patients / all patients)	40 (93%)	33 (91.7)	1.000^d^

^c^Pearson Chi-squared test; ^d^Fisher's exact test

There was no difference between the postoperative de novo urge and stress urinary incontinence rates in the VSSF and LPP groups (Table-4).

The postoperative PISQ-12 scores of the women in the laparoscopy group were significantly higher than those of the women in the vaginal surgery group (Table-5), which means that the postoperative sexual function scores were better in the laparoscopy group. The general scores of the P-QOL questionnaire were not significantly different between the surgery groups. However, there were differences in the general health perceptions, role limitations, and emotion domains between the groups (Table-5).

**Table 5:** Comparison of the Pelvic Organ Prolapse / Urinary Incontinence Sexual Questionnaire (PISQ-12) and Prolapse Quality of Life (P-QOL) scores between the surgery groups.

	Vaginal sacrospinous fixation group	Laparoscopic pectopexy group	p
PISQ-12 score (mean±SD)	36.86±3.15	38.21±5.69	0.029^b^
GHP (mean±SD)	3.93±4.58	4.83±3.33	0.048^b^
PI (mean±SD)	3.09±2.56	2.33±1.76	0.304^b^
RL (mean±SD)	0.09±0.61	0.28±0.70	0.031^b^
PL (mean±SD)	0.14±0.64	0.28±0.61	0.059^b^
SL (mean±SD)	0.14±0.64	0.14±0.54	0.830^b^
PR (mean±SD)	0.51±1.05	0.83±1.27	0.183^b^
EM (mean±SD)	0.14±0.91	0.58±1.02	0.000^b^
SE (mean±SD)	0.09±0.48	0.11±0.32	0.303^b^
SM (mean±SD)	0.12±0.62	0.11±0.39	0.521^b^
GS (mean±SD)	8.49±10.82	9.61±6.99	0.088^b^

**GHP** = general health perceptions; **PI** = prolapse impact; **RL** = role limitations; **PL** = physical limitations; **SL** = social limitations; **PR** = personal relationships; **EM** = emotions; **SE** = sleep / energy; **SM** = severity measures; **GS** = general score

^b^ Mann-Whitney U test

## DISCUSSION

We found that the LPPs and VSSFs were equally effective in the treatment of the uterovaginal / vaginal vault prolapses. The apical descensus relapse rates and patient satisfaction rates were not different.

When the abdominal sacrocolpopexy(ASC) and vaginal surgery results for the apical vaginal prolapsus were compared, the ASC showed better objective anatomic outcomes (8, 9). In addition, the ASC provided better recurrent prolapse rates for 1-2 years and also better objective failure and repeat surgery rates for 2-4 years when compared to the vaginal surgery (10). Although good anatomical and functional results related to sacrocolpexy procedures were reported, new surgical modalities are being applied to more patients every day. Technical difficulties related to the sacrocolpopexy can be seen as due to the difficult surgical field at the ventral site of the sacrum, with a related injury risk to the presacral veins, sacral arteries, and hypogastric nerves. The mesh is usually fixated to the sacral longitudinal ligament, and problems related to the mesh exposure, such as erosion, ileus, and osteomyelitis can be seen (11). Additionally, the decrease in the pelvic space can cause de novo constipation (12).

Pectopexy is an alternative technique to sacropexy, which has certain advantages such as the technique is easy to learn, has a shorter operative time, and has similar positive results (4). Noe et al. compared the postoperative and intermediate-term follow-up results between 44 pectopexy and 41 sacropexy patients. They reported that the apical descensus relapse rates and de novo rectocele rates were no different. In addition, the laparoscopic pectopexy group had lower lateral defect cystocele and de novo constipation rates (12). Moreover, they noticed that the pectopexy had a protective effect on the anterior lateral compartments (12). Although during L / S pectopexy operations vaginal apex is suspended upwards and anteriorly, and the body’s center of gravity deviated to the posterior, postoperative de novo rectocele rates were no different between pectopexy and sacropexy patients. Similarly in the present study, although the vaginal surgery group had more posterior colporrhaphy operations, de novo rectocele rates were no different between VSSF and LPP groups. Moreover in the present study although the vaginal surgery group had more anterior colporrhaphy operations, we found higher de novo cystocele rates in the vaginal sacrospinous fixation group when compared to the pectopexy group. This could be explained by the fact that the vaginal axis in the VSSF group deviated to the right and posterior, and the body’s center of gravity deviated to the anterior, resulting in more weight on the anterior compartment.

It is reported that using mesh in the surgical treatment of cystocele and POP might worsen the sexual function of women in the dyspareunia and behaviour domains (13, 14). Besides, in the study by Vitale et. al., women with severe cystocele were treated with biocompatible porcine dermis graft and they reported a significant improvement in the QoL and sexual function scores 12 months after surgery (14). In our study, the postoperative sexual function of the women in the laparoscopy group was better than that in the vaginal surgery group. This could be related to the scar formation in the vagina after vaginal surgery, vaginal axis deviation to the right and posterior because of unilateral SSF and related sexual problems. Hereof Vitale et. al. evaluated the efficacy and effect of bilateral sacrospinous fixation on QoL and sexuality of women with recurrent vaginal vault prolapse, and they showed a significant improvement in the QoL and sexuality scores (15).

In the current study, we found high patient satisfaction rates (93% in the vaginal surgery group and 91.7% in the laparoscopy group). The general quality of life scores were no different, and they could be accepted as good in both groups, which is compatible with the high patient satisfaction rates. Similarly, Tahaoğlu et al. showed significant improvement in the P-QOL scores pre and postoperatively in the laparoscopic pectopexy group (16). Kale et al. evaluated the short-term outcomes of initial experience with pectopexy surgeries, they reported successful intraoperative and 6. month postoperative results (17). In the present study, although fewer patients in the pectopexy group underwent anti-incontinence surgery, the de novo urge and stress urinary incontinence rates were not different between the groups. This could be attributed to the protective effect of the pectopexy on the anterior lateral compartments (12).

The small number of cases, short term follow-up period and the retrospective design are the major limitations of this study. Since pectopexy procedure has been widely performed in the world since 2010, studies reporting long term results of pectopexy operation with more patients will be published in the following years. Besides VSSF group had more hysterectomies and colporrhaphy operations than LPP group. This could be explained as our hospital is a center that patients are referred from different cities. Most of the women who have completed her fertility and over 45 years want to have hysterectomy because of follow-up difficulties. Another limitation is that, unlike Noe et al. using polyvinylidenfluoride (PVDF) in their original work, we used meshes made of polypropylene, which is widely used in sacrocolpopexy operations (12). The major strengths of this study were that all of the patients (except one, who had relapses and was interviewed over the phone) were gynecologically re-examined postoperatively.

We believe that a pectopexy can be a good choice for prolapse surgery. Surgeons experienced in the laparoscopy and familiar with the pelvic anatomy can easily perform a laparoscopic pectopexy. However, the vaginal sacrospinous fixation maintains its value in prolapse surgery with the increasing importance of native tissue repair. Overall, a pectopexy presents a new, promising, safe method for prolapse surgery. Long-term multicenter follow-up results will help both surgeons and patients to better understand their role in prolapse surgery.
